# Comparison of error correction algorithms for Ion Torrent PGM data: application to hepatitis B virus

**DOI:** 10.1038/s41598-017-08139-y

**Published:** 2017-08-14

**Authors:** Liting Song, Wenxun Huang, Juan Kang, Yuan Huang, Hong Ren, Keyue Ding

**Affiliations:** 1 Key Laboratory of Molecular Biology for Infectious Diseases (Ministry of Education), Institute for Viral Hepatitis, Department of Infectious Diseases, The Second Affiliated Hospital, Chongqing Medical University, Chongqing, 400010 P.R. China; 2 Center for Hepatobillary and Pancreatic Diseases, Beijing Tsinghua Changgung Hospital, Medical Center, Tsinghua University, Beijing, 100044 P.R. China

## Abstract

Ion Torrent Personal Genome Machine (PGM) technology is a mid-length read, low-cost and high-speed next-generation sequencing platform with a relatively high insertion and deletion (indel) error rate. A full systematic assessment of the effectiveness of various error correction algorithms in PGM viral datasets (e.g., hepatitis B virus (HBV)) has not been performed. We examined 19 quality-trimmed PGM datasets for the HBV reverse transcriptase (RT) region and found a total error rate of 0.48% ± 0.12%. Deletion errors were clearly present at the ends of homopolymer runs. Tests using both real and simulated data showed that the algorithms differed in their abilities to detect and correct errors and that the error rate and sequencing depth significantly affected the performance. Of the algorithms tested, Pollux showed a better overall performance but tended to over-correct ‘genuine’ substitution variants, whereas Fiona proved to be better at distinguishing these variants from sequencing errors. We found that the combined use of Pollux and Fiona gave the best results when error-correcting Ion Torrent PGM viral data.

## Introduction

Next-generation sequencing (NGS) has been widely used in the study of viruses and has opened new avenues for research and diagnostic applications (e.g., viral mutant spectra^1, 2^, virus quasispecies theory and dynamics^3–7^, fitness landscape^8, 9^ and discovery of novel viruses^5^). Ion Torrent Personal Genome Machine (PGM) technology is a mid-length read, low-cost and high-speed NGS platform^10^ with special applications in microbial sequencing^11^. However, PGM has a relatively high insertion and deletion (indel) error rate of 1.5% (range from 0.46% to 2.4%)^12–14^.

Several algorithms have been proposed to correct sequencing errors for PGM data (Table 1). These algorithms differ with respect to error models, statistical techniques, data features, the determined parameters, and performances. These methods are classified into the following three categories: (1) suffix array/tree-based methods that use a suffix tree to detect and correct substitution and indel errors (e.g., Fiona^15^); (2) *k*-spectrum-based methods that divide reads into *k*-mer lengths and generate a *k*-mer depth profile to correct the *k*-mer profile (e.g., Blue^16^ and Pollux^17^); and (3) multiple sequence alignment (MSA)-based methods that use *k*-mers as seeds and construct a consensus sequence from the multiple alignments to correct errors (e.g., Coral^18^ and Karect^19^). Two review articles^12, 20^ have systematically surveyed these methods for PGM data and provided guidance concerning which tools to consider for benchmarking based on the data properties. Sequencing data generated in NGS platforms were analyzed in four microbial genomes to assess the coverage distribution, bias, GC distribution, variant detection and accuracy^13^. However, these algorithms have not been fully assessed and applied to viral sequencing data (e.g., hepatitis B virus, HBV).

**Table 1:** Algorithms for error correction in Ion Torrent PGM data.

Method	Algorithm	Comment	Quality score	Input file	Target error type	Ref.
Fiona	Suffix array/tree	Use a suffix tree to detect and correct substitution and indel errors, and use edit distance comparisons to enhance overlap detection of indel errors.	Not used	fasta/fastq	Substitution Deletion/Insertion	15
Pollux	*k*-spectrum	Divide all across reads into *k*-mer lengths, count the observed *k*-mer numbers, and generate *k*-mer depth profiles to correct the *k*-mer profiles. Compare the adjacent *k*-mers and identify discontinuities to find error locations and evaluate correctness.	Not specifically used	fastq	Substitution Deletion/Insertion	17
Blue	*k*-spectrum	Tile reads to reduce the *k*-mer spectrum, distinguish *k*-mers from organisms or containing sequencing error reads, and choose between alternative replacement *k*-mers and a *k*-mer spectrum trust threshold to correct the reads.	Not used	fasta/fastq	Substitution Deletion/Insertion	16
Karect	MSA	Take each read *r* as a reference and perform multiple alignments by selecting optimized reads similar to *r*; represent graph reads; and compute graph edge weights and construct corrected reads.	Not used	fasta/fastq	Substitution Deletion/Insertion	19
Coral	MSA	Compute initial read overlaps with hash tables to the *k*-mer length, form multiple alignments of the reads and rely on quality scores to distinguish and correct erroneous bases.	Used	fasta/fastq	Substitution Deletion/Insertion	18

HBV has a partially double-stranded DNA genome, and its replication depends on reverse transcription of an RNA intermediate by reverse transcriptase (RT). Since the RT lacks proofreading, errors in HBV DNA replication occur at a relatively higher rate than other DNA viruses, with an estimated nucleotide substitution rate of 1.4–3.2 × 10^−5^ substitutions per site per year^21^. Nucleos(t)ide analogs (NAs) have been widely used in anti-HBV therapy by directly inhibiting the HBV RT enzyme and effectively suppressing viral replication^22^. However, long-term use of NAs leads to drug resistance. Characterizing the mutation spectrum and reconstructing the viral quasispecies in the HBV RT region has implications for understanding drug resistance due to NA therapy^23^. For example, various HBV quasispecies associated with drug resistance exist prior to treatment and increase in abundance following anti-viral therapy^24^. Therefore, distinguishing true variants, especially low-frequency mutations, from sequencing errors is crucial for viral mutation-related studies, including quasispecies reconstruction, which is feasible only with the longer 454/Roche reads^25^.

In the present study, we investigated the performance of error correction algorithms in empirical and simulated PGM data for the HBV RT region. We have the following aims: 1) to characterize the error profiles of 19 quality-trimming PGM datasets for the HBV RT region; 2) to assess the error-correction performance of algorithms in empirical and simulated data under different models; and 3) to provide a benchmark for generating an analysis-ready alignment of PGM data for studies of viral sample sequencing.

## Results

### Summary of empirical datasets

Using the Ion Torrent PGM platform, we sequenced the extended HBV RT region (~1300 bp) in 19 viral samples. A summary of the sequencing data is presented in Table 2. After quality-trimming the original reads, an average of 18.6% of the reads were filtered, and 99.76% of the filtered reads were mapped to the sample-specific reference sequence (obtained via Sanger sequencing) with an average coverage of 8,648×. The mean base quality of the filtered reads was 28.

**Table 2:** Summary of the Ion Torrent PGM data for the HBV RT region.

ID	Total reads	Read length (mean ± sd)	Average depth	Reads removed (%)	Mapped rate (%)	Mean base quality
009	44,240	282 ± 51	10,198	42	99.98	28
014	52,039	268 ± 68	8,484	24	99.86	27
017	61,174	267 ± 64	10,481	20	99.98	27
020	42,097	259 ± 73	7,017	32	99.95	26
024	56,729	268 ± 67	10,139	21	99.93	27
033	55,425	264 ± 74	9,807	25	99.97	27
037	39,971	268 ± 76	7,255	24	99.97	26
040	35,191	287 ± 79	7,145	27	100.00	26
042	41,108	282 ± 76	8,384	26	99.95	26
1005	40,081	299 ± 78	8,362	12	99.86	28
1009	32,042	326 ± 75	7,368	16	99.99	28
1014	36,652	306 ± 63	7,526	12	99.90	28
1019	44,490	292 ± 71	8,108	12	96.75	29
1024	57,965	290 ± 64	10,793	9	99.89	29
1028	32,852	290 ± 63	6,126	10	99.95	29
1034	42,626	293 ± 62	8,490	10	99.99	29
1038	49,963	308 ± 62	9,972	12	99.87	29
1041	40,300	314 ± 66	7,764	12	99.83	29
1046	59,393	298 ± 65	10,900	9	99.92	28

### Estimation of the error rate in the empirical PGM data

The quality-trimmed reads were aligned to the sample-specific reference sequence using the Torrent Mapping Alignment Program (TMAP). The pre-correction alignment was analyzed using the Error Correction Evaluation (ECE) Toolkit^20^ to generate the target error format (TEF) file. The base-error rate (*R*_*error*_) was empirically estimated for different types and regions using

1 $${R}_{error}=(\sum _{i=1}^{n}{r}_{i})/(\sum _{i=1}^{n}nbas{e}_{i})$$

where *r*_*i*_ was the number of errors in each read (*i*), *nbase*_*i*_ was the total number of sequenced bases, and *n* was the total number of reads. For example, the deletion error rate in the homopolymers was calculated by dividing the total number of deletion errors by the total number of sequenced bases in the homopolymer region. A homopolymer region was defined as a homopolymer repeat with a length ≥*hl*, where 2 ≤*hl* ≤5. This definition was established to ensure that indel errors, which were common on this platform, were truly reflected by the error rate. To estimate the substitution error rate, we excluded the defined ‘genuine’ mutations (i.e., a variant with a frequency ≥0.5% based on the TEF file from the pre-corrected alignments), because Ion Torrent PGM could detect substitutions occurring at frequencies ≥0.1%^26^.

**Figure 1:**
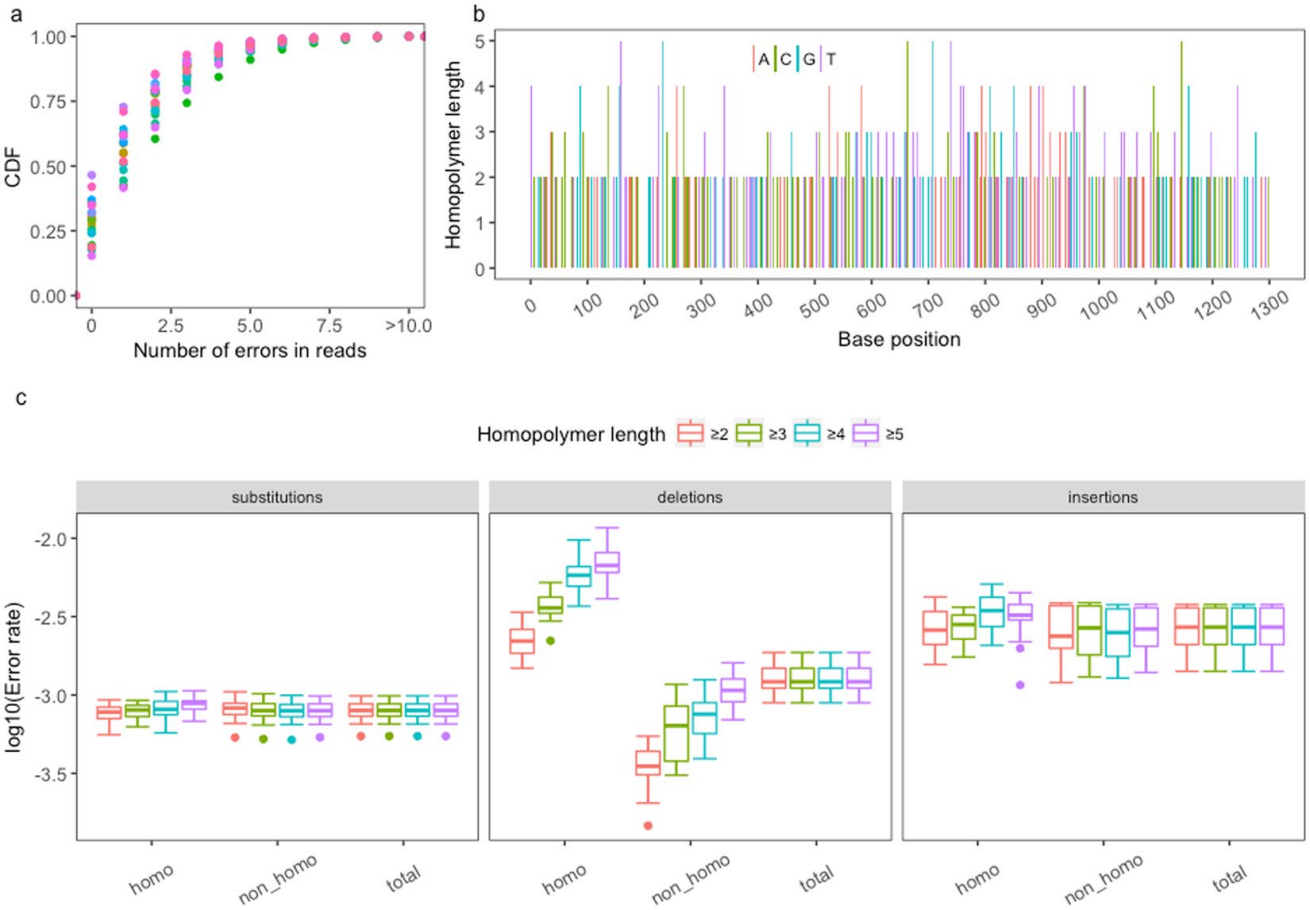
Error profiles of the Ion Torrent PGM data for the 19 raw samples. (**a**) Cumulative density of errors in the sequencing reads in the 19 sequencing data sets. Different colors indicate different samples. (**b**) Distribution of homopolymers with different lengths in the RT region of an HBV reference genome (AB033556). (**c**) Estimation of the error rates of insertions, deletions, and substitutions in 19 PGM data sets for the HBV-RT region grouped by regions (homopolymer, non-homopolymer and total).

The cumulative distribution of errors in the sequencing reads after quality trimming indicated that 99.48% of the sequencing reads had ≤9 errors (Fig. 1a). We did not find any ‘true’ indels using Sanger sequencing; therefore, all indels can be considered errors. The distribution of homopolymers with different lengths (2 ≤*hl* ≤5) in the HBV RT region (AB033556) is shown in Fig. 1b. We counted the numbers of each type of error in the homopolymer and non-homopolymer regions. Insertion and deletion errors occurred more frequently than substitution errors (Fig. 1c). Notably, deletion errors were more likely for homopolymers and were correlated with *hl*. When *hl* ≥ 4, the mean deletion error rate in the homopolymers was 0.59%, although the insertion error rate (0.27%) was more likely to be greater than the deletion error rate (0.13%) in the total sequenced regions. As noted previously, PGM data were sensitive to homopolymers^13^, and the indel error rate increased as *hl* increased^27, 28^.

### Comparison of error correction algorithms using empirical PGM data

Several measures have been proposed to evaluate the quality of error correction^29^, including the measure of gain, sensitivity and specificity. The gain

2 $$gain=(TP-FP)/(TP+FN)$$

is a widely used measure^19, 20, 29, 30^ that is equivalent to the number of true errors corrected (TP) minus the number of introduced errors (FP) divided by the total number of errors initially present in the data (TP + FN)^20^. This measure penalizes failing to detect an erroneous base, correctly detecting but wrongly correcting an erroneous base, and characterizing a correct base as an erroneous base^31^. Generally, real sequencing errors were obtained by mapping the sequencing reads to the reference genome and recording the differences. When both substitution and indel errors were targeted for correction, the TP, FP and FN were inferred as follows^20^. The algorithm defines *r* as an original read and *rc* as the read post-correction. The set of real sequencing errors (*E*_*m*_) is derived by mapping *r* to the reference and recording the differences, and the set of errors remaining in *rc* (*E*_*r*_) is measured by applying a global alignment between *rc* and the genomic region to which *r* is mapped and recording the differences in the alignment. Accordingly, TP, FP, and FN are calculated as: $TP=|{E}_{m}\backslash {E}_{r}|$, $FP=|{E}_{r}\backslash {E}_{m}|$ and $FN=|{E}_{r}\cap {E}_{m}|$.

**Figure 2:**
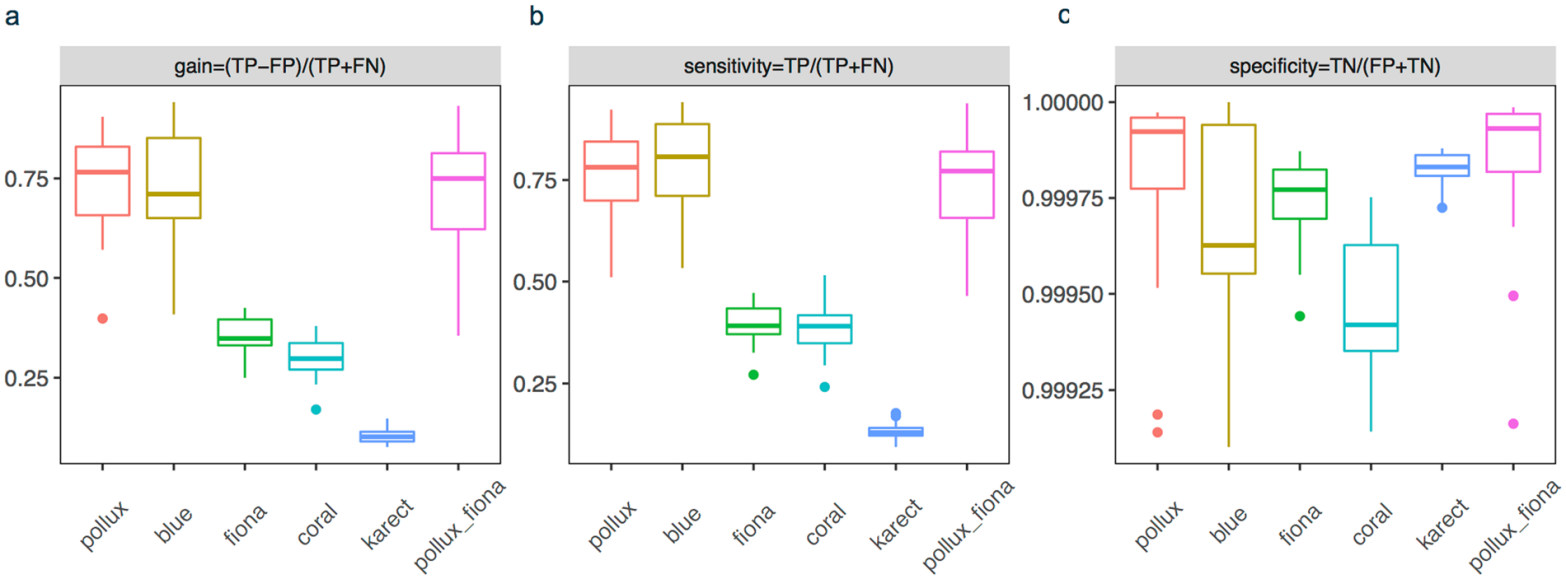
Comparison of the error correction algorithms using 19 empirical data sets sequenced in the HBV RT region with respect to the measure of gain (**a**), sensitivity (**b**), and specificity (**c**).

On average, 0.48% and 8.21% of the reads were discarded by Pollux and Blue, respectively (Table S1). The error correction performance in the 19 PGM data sets differed significantly among the algorithms (Fig. 2). The measures of gain obtained by Pollux (mean of 0.74) and Blue (0.60) were significantly greater than the measures of gain obtained by Fiona, Coral and Karect (ANOVA, *p* = 3.41 (3.45) × 10^−14^, *p* = 1.24 (1.24) × 10^−14^, and *p* = 1.15 (1.15) × 10^−14^, respectively). The sensitivity of the five algorithms appeared to be similar to the measure of gain (Fig. 2b), and the specificity was similar (>99.5%) (Fig. 2c). A negative correlation was found between the measure of gain and the residual error rate of the post-corrected reads (*r* = −0.8) (Table S2).

**Figure 3:**
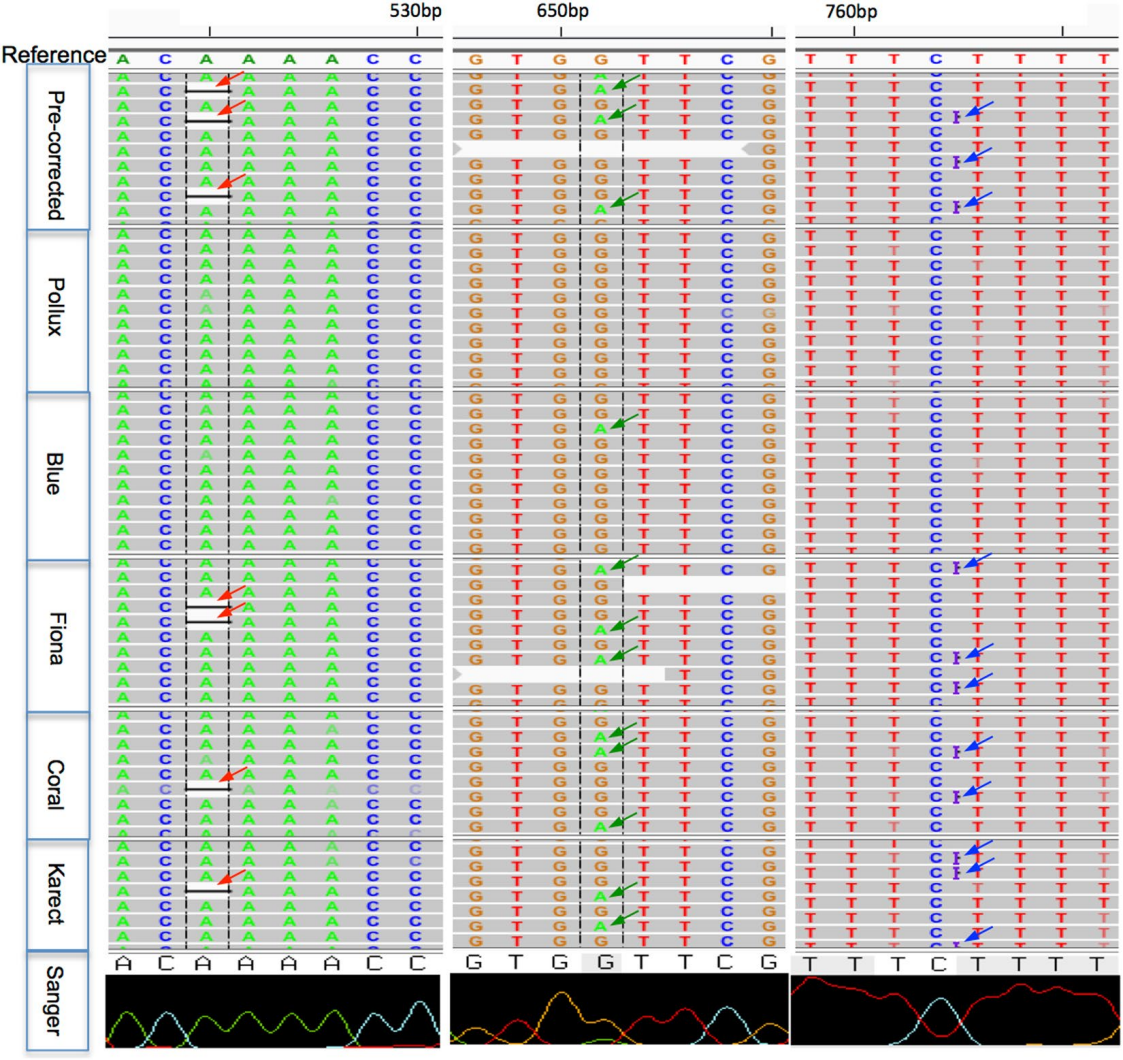
A read alignment view at an 8-bp resolution for error-correction using different algorithms. Arrows in different colors represent different types of errors or true mutations. Red: deletion errors; green: true substitution mutations; and Blue: insertion errors.

We manually investigated the behaviors of these algorithms in correcting for insertion (blue arrow), deletion (red arrow) and substitution (green arrow) errors (Fig. 3). We found that Pollux and Blue had a greater power for indel error correction but were unable to distinguish ‘genuine’ substitutions from errors. For example, at position 651 (a G → A Sanger-confirmed mutation), most of the mutated ‘A’ alleles (959 out of 7427) were falsely corrected by Pollux (956/959) and Blue (788/959) but not by Fiona, Coral and Karect. For the insertion errors between positions 762 and 763 (1,070 out of 7,208 sequencing reads), Pollux and Blue corrected 98.2% and 100% of the erroneous insertions, followed by Coral (25.3%), Fiona (1.3%) and Karect (0%). We noted similar behaviors of these algorithms for deletion error (e.g., at position 525) corrections.

**Figure 4:**
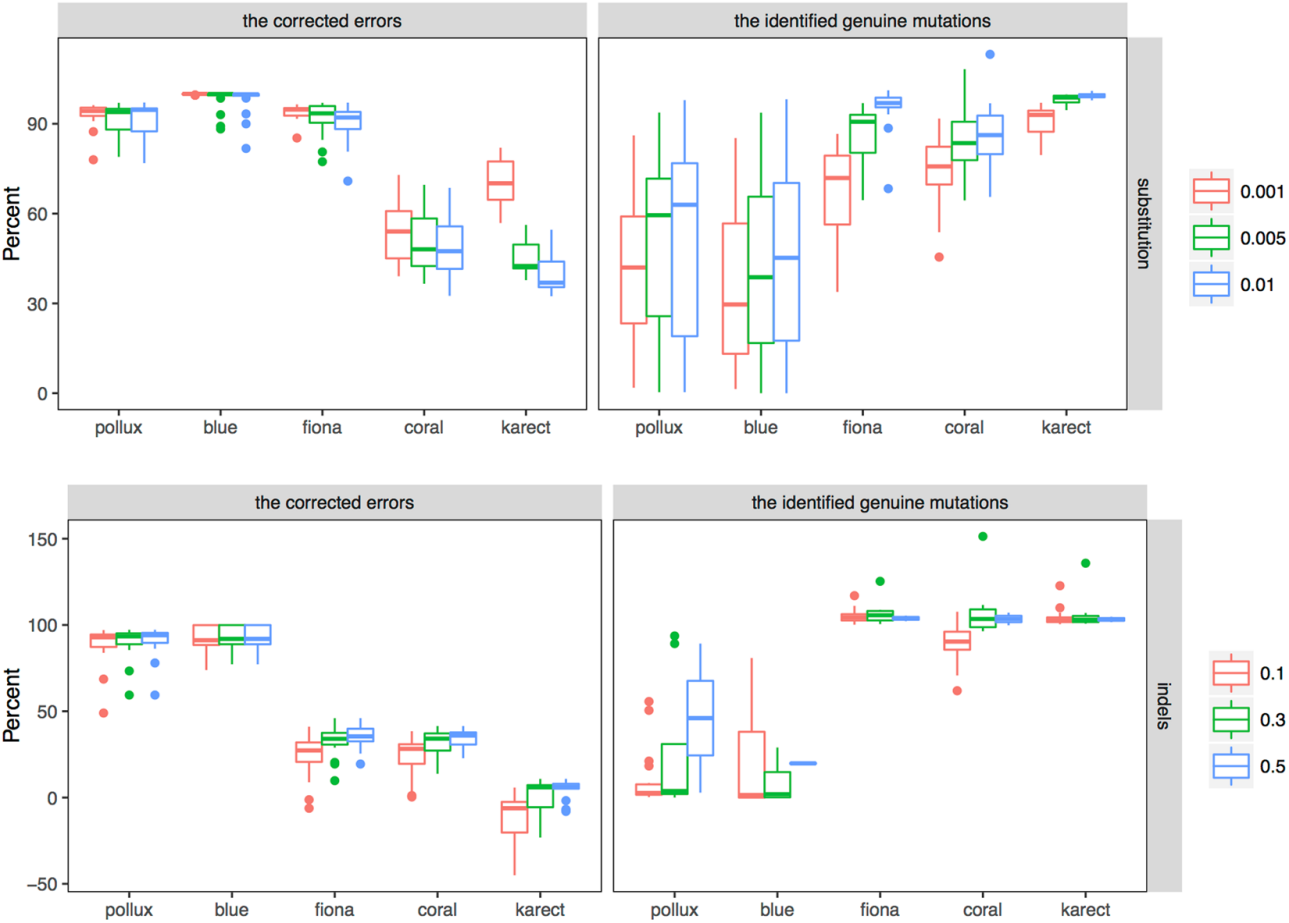
The proportion of the identified ‘genuine’ mutations and the corrected errors under different algorithms based on the pre- and post-corrected TEF files in the empirical PGM data.

The ECE toolkit takes all bases differing from the reference as errors and counts all corrections changed to the reference as a TP, resulting in a bias in the calculation of these measures. We set different frequency thresholds (0.1%, 0.5% and 1%) to distinguish ‘genuine’ substitutions and errors, because Ion Torrent PGM can detect substitutions occurring at frequencies ≥0.1%^26^ (i.e., a variant was considered to be ‘true’ if its frequency was greater than the cutoff). Based on the pre- and post-corrected TEF files, we counted the proportion of the identified ‘genuine’ mutations and the corrected errors under different algorithms (Fig. 4). We calculated the proportion of the identified ‘genuine’ mutations by dividing the number of mutated alleles in the corrected reads by the number in the original reads. We found that Pollux and Blue over-corrected for ‘genuine’ substitutions with a higher frequency, whereas Karect and Coral had a lower power for error correction. Fiona corrected most of the substitution errors with frequencies <1% and preserved the variants with relatively higher frequencies. However, this algorithm had limited power for correcting indels, which are the main type of errors in the Ion PGM data. The greater gain of Pollux may be due to its power for indel error correction as well as its effect on falsely correcting ‘genuine’ substitutions. Therefore, we suggest the combination of Pollux (for indel error correction only) and Fiona (for substitution error correction) for Ion Torrent PGM data (Pollux_Fiona). The measures of gain (*p* = 0.79), sensitivity (*p* = 0.52), and specificity (*p* = 0.35) obtained by Pollux_Fiona did not differ significantly from the measures obtained with Pollux (Fig. 2).

We also changed the *k*-mer parameter to optimize the *k*-spectrum-based algorithms (Blue and Pollux) and the MSA-based method using *k*-mer (Coral) for error correction. The measure of gain did not differ significantly under different *k*-mer values (ANOVA, *p* = 0.45 (Pollux) and 0.20 (Coral)) but was marginal in Blue (*p* = 0.04) (Fig. S1). The average time costs for Pollux, Blue, Fiona, Coral, and Karect were 5.2, 2.2, 36.1, 18.6 and 1.2 minutes, respectively, showing that Fiona was the most time-consuming algorithm.

### Performance of error correction algorithms using simulated data

**Figure 5:**
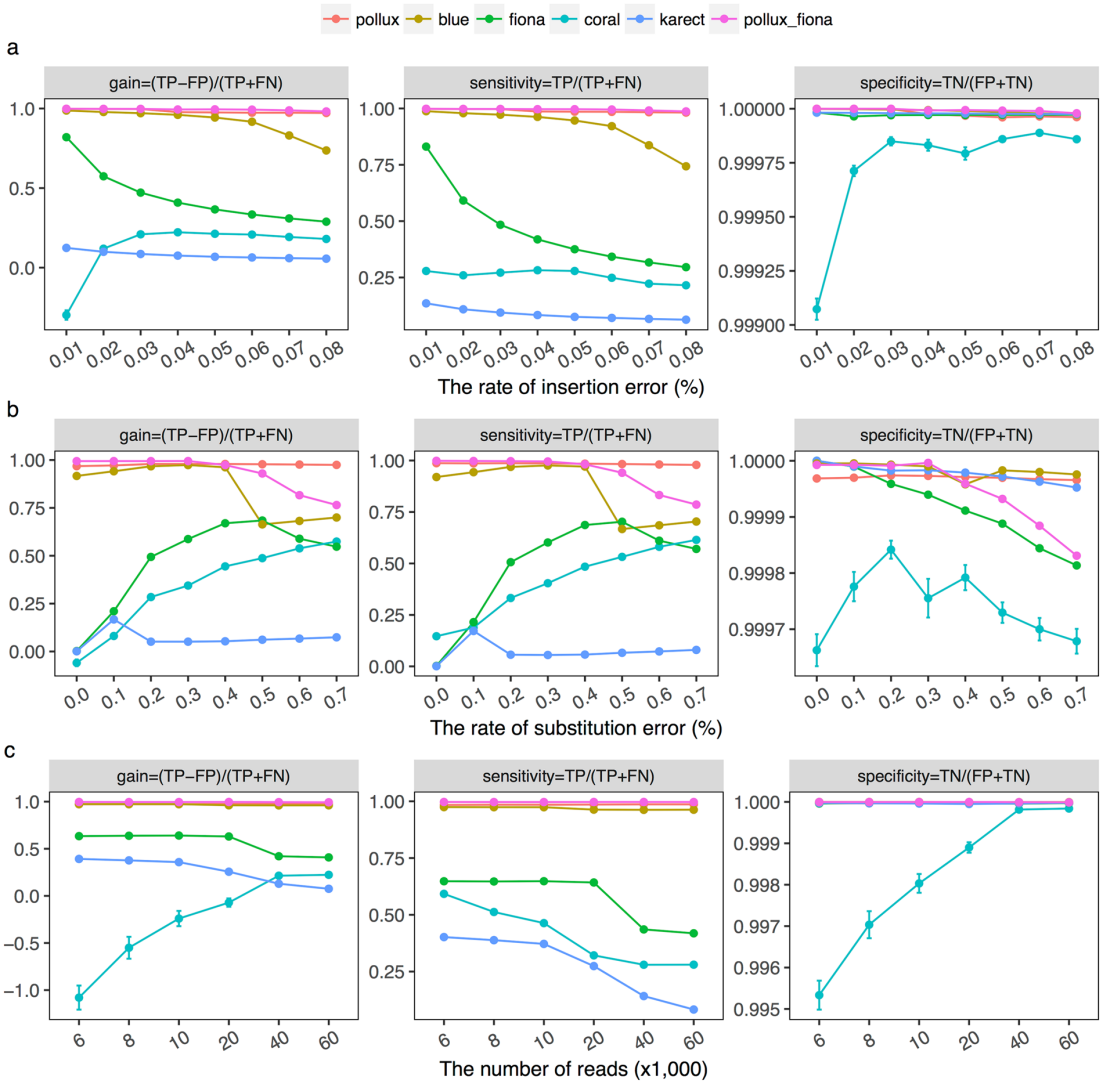
Error correction performance in the simulated PGM data. (**a**) A model of indel errors. We assumed a fixed substitution rate (0.17%) and read number (60,000) with varied indel error rates, and the deletion error rate was 1.5 times the insertion error rate. (**b**) A model of substitution errors. We assumed a fixed insertion (0.04%) and deletion (0.06%) error rate and read number (60,000) with varied substitution error rates; and (**c**). A model of sequencing depth. We assumed fixed insertion (0.04%) and deletion (0.06%) error rates and substitution error rates (0.17%) with different sequencing depths.

We studied the performance of the different algorithms under different simulation scenarios. First, a model of indel errors (Fig. 5a) showed that the measures of gain differed significantly among these algorithms (ANOVA, *p* < 2.2 × 10^−16^) and the indel error rates (*p* = 2.1 × 10^−8^). In concordance with the empirical data, Pollux had a better performance in measure of gain (~1) and remained similar with an increased indel error rate; Blue exhibited similar behavior, but its performance decreased when the insertion error rate was ≥0.06 or the deletion rate was ≥0.09. Fiona showed a relatively higher measure of gain in the setting of an error rate ≤0.02, since most of the substitution errors (the major errors under this setting) were removed. The introduction of a large number of insertion errors (rate of 0.01) at the homopolymer regions (*hl* ≥ 5) resulted in a negative gain for Coral and a relatively lower specificity. Both Fiona and Coral obtained moderate measures of gain and sensitivity with an increased error rate. Karect had a lower performance for correcting indel errors regardless of the rate. As expected, the combined use of Pollux and Fiona had a similar performance with Pollux.

Second, we investigated the effects of the substitution errors for the performance (i.e., a model of substitution errors) (Fig. 5b). Similarly, Pollux out-performed the remaining algorithms under different rates. However, Karect obtained a higher measure of gain when the substitution rate was 0.1% partly due to its effects in correcting for low-frequency substitution errors. Obviously, Blue, Fiona and Coral had better performances at higher substitution rates, but the performances of Blue and Fiona decreased as the errors continued to accumulate. Blue had an especially good performance when the substitution error rate was ≤0.4%, but its power for error correction decreased significantly when the rate was ≥0.4%. We speculated that the enrichment of errors in reads might have a significant effect on the *k*-mer count profile and error inference. We also simulated a set of data by randomly introducing known variants into the reads, including five rare mutations (with frequencies of 0.1–0.5%) and three low-frequency variants (approximately 5%). The proportion of the remaining mutated alleles and sequencing errors after error correction (Table S3) indicated that Pollux and Blue could not distinguish rare and low-frequency variants from sequencing errors, whereas Fiona could identify low-frequency variants but not rare mutations. Although Coral and Karect could identify the rare and low-frequency variants, these algorithms had little power for sequencing error correction. These results were consistent with our analyses of the empirical data (Fig. 4).

Finally, we explored how the sequencing depth affected the performance (i.e., a model of the sequencing depth). The sequencing depth had little effect on Blue and Pollux (Fig. 5c), whereas Fiona and Karect exhibited a better performance with a lower depth. However, Coral obtained a negative measure of gain under a lower depth (e.g., 6,000 reads), probably resulting from a higher FP introduced by insertion errors. The combined use of Pollux and Fiona had a similar performance as Pollux.

## Discussion

Relatively higher mutation and replication rates in viruses lead to an increased number of mutations, including a large number of rare variants. Ultra-deep sequencing has been widely used for analyses of viral populations^32, 33^ and enables the examination of the diversity of the whole viral population and the identification of important variants present within the viral population at low frequencies (i.e., mutations that increase pathogenicity or convey drug resistance^33^). Therefore, the characteristics of viral sequencing data include a higher sequencing depth and a broad frequency spectrum of mutations compared with sequencing data for macro-organisms. Therefore, effectively distinguishing low-frequency variants from sequencing errors remains a great challenge.

Bragg *et al*.^26^ described the biases and errors introduced by PGM across a combination of factors in two bacterial species. The average GC content of *Bacillus amyloliquefaciens* (46%)^26^ is similar to the empirical (49.9%) and simulated data (46.4%) in our study. The authors found indel errors at a rate of 1.38% after quality clipping, which accounted for most of the errors due to inaccurate flow calls. In our PGM data, the deletion errors in the homopolymers (i.e., a polymer consisting of ≥4 identical nucleotides) were significantly greater than those in the non-homopolymers, but the insertion error rate was not increased in the homopolymers (Fig. 1c). The adaptor may increase the error rate of Ion Torrent PGM data^34, 35^; however, the final error rate of Ion Torrent PGM sequencing of all chips was approximately 1% (range from 0.46% to 2.4%)^14^ (http://www.molecularecologist.com/next-gen-fieldguide-2016). The total error rate in our original reads was 0.61% ± 0.16%, but this rate decreased after quality trimming (0.48% ± 0.12%). The difference in the estimated error rate may be partly due to differences in template preparation, the use of a different sequencing kit, and different species.

Of these correction algorithms, we noted different performances in both the empirical and simulated PGM data (Figs 2 and 5). Generally, Pollux and Blue had similar performances, and their measures of gain were significantly greater compared to the remaining algorithms, which was consistent with previous studies^16, 17^. There are several explanations for their ‘outperformances’. First, Pollux and Blue filter and discard reads that appear to still be faulty after correction (averages of 0.48% and 8.21%, respectively, in our 19 PGM data sets). Second, Pollux performs homopolymer corrections independently after exhausting all other correction possibilities^17^. Third, both algorithms over-corrected for the ‘genuine’ substitutions (Figs 3, 4 and Table S3) (e.g., more than 97% of the mutated alleles of the variants with an approximate frequency of 5% were falsely corrected). Of the remaining algorithms, Fiona had a greater measure of gain than Coral, which was consistent with Schulz *et al*.^15^, where Fiona showed a higher correction accuracy over a broad range of datasets from 454 and Ion Torrent sequencers and outperformed Coral. Fiona seemed to have a greater power for distinguishing ‘genuine’ substitutions with a relatively higher frequency from errors but a limited power for indel correction (Figs 3 and 4 and Table S3). Allam *et al*.^19^ showed that Karect was more accurate than the other methods (e.g., Fiona, Blue and Coral) in terms of correcting single base errors (up to a 10% increase in gain). Our results indicated that Karect had little power for indel error correction (Fig. 5a) with the exception of a low substitution error rate and sequencing depth (Fig. 5b and c). In summary, sequencing for different species (i.e., eukaryotes, prokaryotes or viruses), the sequencing depth, and error profiles in different platforms may influence the error-correction performance. Since Pollux has a greater performance for indel error correction and Fiona has a greater power for distinguishing ‘genuine’ substitutions from sequencing errors, we suggest the combined use of Pollux and Fiona for Ion Torrent PGM data (Pollux_Fiona).

The present study has several limitations. First, simulating sequencing reads of substitutions with different frequencies and introducing sequencing errors will clarify whether error correction can be used to reduce ‘genuine’ errors and leave low-frequency variants alone. The substitution errors simulated by ‘CuReSim’ followed an exponential distribution with an increased probability of occurring at the end of the reads, and the error direction was random. Second, we acknowledge a potential lack of robustness in distinguishing ‘genuine’ mutations and errors based only on the defined frequency thresholds (Fig. 4), and ‘genuine’ rare mutations may have been present with frequencies less than the given threshold. The simulated data with known variants indicated that these algorithms could not distinguish rare variants from substitution errors (Table S3). Finally, we did not identify ‘genuine’ Sanger-confirmed indels in our HBV sequencing data. We suggest that the use of Pollux may remove the predominant indel errors introduced by poor handling of short homopolymer runs in Ion Torrent. However, as shown in Fig. 4, Pollux and Blue over-corrected the defined ‘genuine’ indels even with a frequency greater than 50%. Therefore, the potential for over-correction of indels cannot be ignored if ‘genuine’ indels exist, which is a common phenomenon in viruses^36^.

In conclusion, we provided a benchmark for error correction algorithms that can be used in PGM data applications for viral genome sequencing. We suggested the combined use of Pollux and Fiona as a better choice for its performance in both the real HBV Ion Torrent PGM and simulated data. However, vigorous algorithms need to be developed for PGM data in the setting of distinguishing low-frequency variants and sequencing errors.

## Methods

### Empirical data

#### Chronic hepatitis B virus (HBV)-infected patients

Patients with chronic HBV infection were recruited from the Department of Infectious Diseases, the Second Affiliated Hospital of Chongqing Medical University, Chongqing, China. None of the patients were receiving oral nucleoside/nucleotide analogues (NAs) or interferon-alpha (IFN-*α*) antiviral therapy. The study was approved by the Institutional Review Board (IRB) of the Second Affiliated Hospital of Chongqing Medical University, and the patients provided written informed consent. All of the experiments were performed in accordance with the relevant guidelines and regulations.

#### HBV DNA extraction and RT region amplification

In total, 19 serum samples collected at baseline were obtained in the present study. HBV genomic DNA was extracted using the QIAamp UltraSens^TM^ Virus Kit according to the manufacturer’s protocol. A nested PCR was performed to amplify the HBV RT regions^37^, and the PCR products were purified using the QIAquick PCR Purification Kit (Qiagen^**®**^, Hilden, Germany).

We used the DNA fragmentation strategy to construct shotgun fragment libraries for Ion Torrent PGM sequencing to produce shotgun reads with a target fragment size range of 200–400 bp^38^. Library preparation was conducted using the Ion Xpress Plus Fragment Library Kit (Cat. no. 4471269, Pub. no. MAN0009847, Rev. C) with 100 ng of HBV DNA. Adapter ligation, size selection, nick repair and amplification were performed according to the manufacturer’s protocol. Sample emulsion PCR, emulsion breaking and the enrichment steps were performed using the Ion PGM Template OT2 400 Kit and the associated protocol (Cat. no. 4479878, Pub. no. MAN0007218, Rev. A) according to the manufacturer’s instructions. Briefly, an input concentration of template-positive Ion Sphere Particles (ISPs) was added to the emulsion PCR master mix to generate the emulsion. After enriching template-positive ISPs, sequencing was undertaken using the Ion 318 Chip v2 in the Ion Torrent PGM System. The Ion PGM Hi-Q Sequencing Kit was used for all sequencing reactions according to the protocol (Cat. no. A25592, Pub. no. MAN0009816, Rev. D). All PGM sequencing was conducted by the WuXi AppTec company (Shanghai, China).

PCR chimeras are common in amplicon sequencing where closely related sequences are amplified but are rare with shotgun sequencing. In the setting of next-generation sequencing, the formation of artificial chimeras during PCR can be consistently suppressed to low levels^39^. We used the DNA fragmentation strategy to construct shotgun fragment libraries for Ion Torrent PGM sequencing. Therefore, the chimeras from PCR may have little or no influence on the HBV PGM data in our study.

#### Sanger sequencing

Standard Sanger sequencing reactions were electrophoresed using an Applied Biosystems 3730xl DNA Analyzer (Applied Biosystems, Foster City, CA, USA). Direct sequencing of the PCR products of the HBV RT region was performed in both directions. Sample-specific reference sequences for the HBV RT region were assembled using Sanger sequencing reads with manual finishing.

### Data simulation

**Figure 6:**
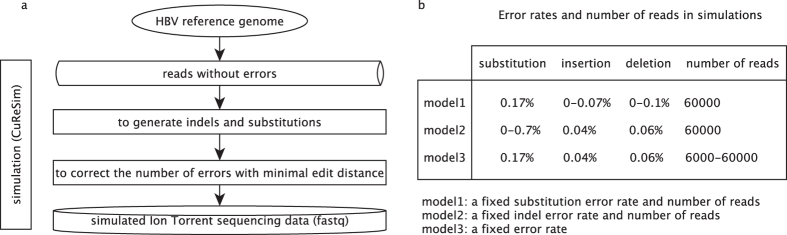
A framework for simulating PGM data. (**a**) Steps for simulating PGM data based on a reference genome using ‘CuReSim’^40^. (**b**) A series of simulation scenarios with a fixed substitution error rate (model 1), a fixed indel error rate (model 2), and a fixed error rate (model 3).

We used the sequence-read simulator program ‘CuReSim’^40^ to generate *in silico* PGM data (Fig. 6a). First, we supplied an HBV reference sequence (AB033556) for the simulation to generate error-free reads given the mean and standard error of the read length obtained from our empirical PGM data. Second, indels and substitution errors were introduced based on a specified error rate. The substitution errors follow an exponential distribution depending on the read position (i.e., the substitution probability increases at the end of the read). Additionally, an iterative algorithm introduced indels in the longer homopolymers^40^. In the empirical data, the number of bases in the total region (${N}_{all\_base}$) was approximately ten times the number of bases in the homopolymer regions (${N}_{homopolymer\_base}$) (e.g., the total number of indel errors (${N}_{all\_indel}$) was approximately equal to the indel errors in the homopolymer regions (${N}_{homopolymer\_indel}$)). The indel error rates ($P{R}_{indel}$) can be estimated by:

3 $$P{R}_{indel}={N}_{all\_indel}/{N}_{all\_base}={N}_{homopolymer\_indel}/{N}_{all\_base}={N}_{homopolymer\_indel}/(10\times {N}_{homopolymer\_base})$$

Therefore, for simulation using ‘CuReSim’, the indel error rate ($P{R}_{indel}$) was approximately one-tenth of the indel error rate in the homopolymer regions (${R}_{homopolymer\_indel}$/10). In our 19 quality trimming PGM data sets, the insertion and deletion error rates in the homopolymer regions were approximately 0.4% and 0.6%, respectively. Therefore, we fixed the error rates of 0.04% and 0.06% (in model 2) to explore the effect of substations on the performances of the algorithms. The re-estimated indel error rates based othe simulated data were similar to the empirical indel error rates. Finally, the number of iroduced errors was corrected using the errors corresponding to the minimal edit distance.

We simulated three models of PGM sequencing reads (Fig. 6b). In the indel error model (Table S4), a set of indel error rates was used given a fixed substitution error rate in the PGM data (0.17%)^12^. The specified indel error rate enabled a similar indel error rate range to be obtained from our PGM data. In the substitution error model (Table S5), we assumed fixed insertion and deletion error rates (0.04% and 0.06%, respectively), and the substitution rate varied from 0 to 0.7%. In the sequencing depth model (Table S6), we simulated a pool of 80,000 reads given a fixed insertion and deletion error rate (0.04% and 0.06%) and a substitution error rate of 0.17%. We down-sampled the pool to generate different numbers of reads from 6,000, 8,000, 10,000, 20,000, 40,000, and 60,000, corresponding to an approximate depth of 1,230×, 1,650×, 2,050×, 4,100×, 8,200×, and 12,300×, respectively. The parameters of the three models are described in detail in the supplementary material (Tables S4–S6).

### Bioinformatics analysis

We used a pipeline to process the empirical or simulated PGM data, including pre-processing, error correction, alignment, and assessment of error correction (Fig. S2).

#### Pre-processing

The empirical raw fastq data were filtered using the ‘fastq_quality_filter’ in the FASTX-Toolkit (http://hannonlab.cshl.edu/fastx_toolkit/). Low-quality reads were filtered if 20% of the bases had a phred quality score <20.

#### Error-correction algorithms

The five algorithms assessed in the present study (Table 1) could take a ‘fastq’ file as input, and the default setting was used when running each program. The command lines for executing these programs were provided in the supplementary material.

#### Alignment

The pre- and post-corrected PGM sequencing reads were aligned to a sample-specific reference sequence (for the empirical data) and AB033556 (for the simulated data) using the Torrent Mapping Alignment Program (TMAP, https://github.com/iontorrent/TS/tree/master/Analysis/TMAP). TMAP uses a series of algorithms (BWA, BWASW, SSAHA2, the super-maximal exact matching algorithm, and the Smith–Waterman algorithm) to map data to an indexed reference sequence. The alignment was performed in two stages with the option ‘mapall -g 0 stage1 map1 stage2 map2 map3’. This process enabled an alignment using BWA^41^ in the first stage (map1) and BWA-SW^42^ (map2) and SSAHA^43^ (map3) in the second stage. Since no known indels were previously reported in the HBV RT region, we did not perform a realignment around indels.

#### Assessment of error correction

The measure of gain, TP, FP, and FN were calculated using the ‘compute-stats.py’ script from the Error Correction Evaluation (ECE) Toolkit (http://aluru-sun.ece.iastate.edu/doku.php?id=ecr)^20^. The command lines used to execute the assessment are provided in detail in the supplementary material.

All calculations were executed using an IBM server with 4x Intel(R) Xeon(R) CPU E7-8850@2.00 GHz processors and 256 GB of memory.

### Data availability

The 19 Ion Torrent PGM sequencing data for the HBV RT region have been uploaded to the NCBI Sequence Read Archive (SRA) (accession number: PRJNA335918). The simulated data were generated using the simulator ‘CuReSim’^40^ and were provided in the Supplementary materials.

## Electronic supplementary material

Supplementary information
