# Supplementary material for: Comparison of error correction algorithms for Ion Torrent PGM data: application to hepatitis B virus
Source: Sci Rep. 2017 Aug 14;7:8106. doi: 10.1038/s41598-017-08139-y (PMC5556038; doi:10.1038/s41598-017-08139-y)
Supplement: Supplementary file 1 — Supplementary information [file 41598_2017_8139_MOESM1_ESM.pdf]

Comparison of error correction algorithms for Ion Torrent PGM data: application to hepatitis B virus

Liting Song[1,3], Wenxun Huang[1,3], Juan Kang[1,3], Yuan Huang[2], Hong Ren[1], Keyue Ding[1,*]

[1]Key Laboratory of Molecular Biology for Infectious Diseases (Ministry of Education), Institute for Viral Hepatitis, Department of Infectious Diseases, The Second Affiliated Hospital, Chongqing Medical University, Chongqing, 400010 P.R. China

[2]Center for hepatobillary and pancreatic diseases, Beijing Tsinghua Changgung Hospital, Medical Center, Tsinghua University, Beijing, 100044 P.R. China

[3]These authors contributed equally

*: Corresponding author

Keyue Ding, Ph.D.

74# Linjiang Men, Yuzhong District, Chongqing, 400010 P.R. China

Phone: +86 23 6389 2759

E-mail: dingkeyue@hospital.cqmu.edu.cn

Supplementary Information

Supplementary materials and methods

1. Generation of Target Error Format (TEF) file. We used 'sam-analysis.py' in Error Correction Evaluation (ECE) Toolkit[1] to generate this file.

python sam-analyis.py --file=${sam file} --outfile=${output file} --ambig=${ambig output} --unmapped=${unmapped file} --trim=${trim file} --genome=${reference genome}

- ${sam file}: the TMAP mapping sam file without error correction
- ${output file}: mapping reads
- ${ambig output}: ambiguous reads
- ${unmapped file}: unmapped reads
- ${trim file}: trimming reads

2. Data simulation. We used the Customized Read Simulator (CuReSim, ver.1.2, http://www.pegase-biosciences.com/curesim-a-customized-read-simulator/)[2] for simulating Ion Torrent PGM data.

java -Xmx20480m -jar CuReSim.jar -f ${reference} -m ${mean of read lengths} -sd ${sd of read lengths} -d ${deletion rate} -i ${insertion rate} -s ${substitution rate} -n ${read number}

- ${mean of read lengths} and ${sd of read lengths}: the mean (267) and standard deviation of read lengths (85)
- ${deletion rate}: deletion error rate
- ${insertion rate}: insertion error rate
- ${substitution rate}: substitution error rate

3. Use of error-correction algorithms. We used uniform parameter values for the five error correction softwares. If some parameters do not alter by the user, then we use default parameter values for the tools.

3.1. Karect[3] (v1.0, http://aminallam.github.io/karect/)

karect -correct -threads=${number of threads} -matchtype=edit -celltype=haploid -inputfile=${input file} -resultdir=${result dir}

- matchtype: is set to "edit" for our datasets is Ion Torrent datasets

- celltype: is set to "haploid" for our samples are hepatitis B virus serum

3.2. Fiona[4] (v0.2.4, http://packages.seqan.de/fiona/)

fiona -g ${genome size} -nt ${number of threads} ${input file} ${result file}

- ${genome size}: the genome size is the size of HBV reverse transcriptase region
- ${input file}: the fastq files of our empirical and simulation data, the same as the other tools
- ${result file}: specify a result fastq files of our empirical and simulation data, the same as the other tools

3.3. Pollux[5] (v1.0.2, *https://github.com/emarinier/pollux*)

pollux -i ${input file} -k ${*k*-mer size} -o ${result dir}

- ${k-mer size}: 31

3.4. Coral[6] (v1.4.1, https://www.cs.helsinki.fi/u/lmsalmel/coral/)

coral -p ${number of threads} -k ${k-mer size} -fq ${input file} -454 -o ${result file}, where ${*k*-mer size} is 21.

3.5 Blue[7] (v1.1.3, http://www.bioinformatics.csiro.au/blue), includes three steps as indicated below:

3.5.1) mono Tessel.exe -k ${*k*-mer size} -g ${genome size} -t ${number of threads} -f fastq ${input file}

3.5.2) mono GenerateMerPairs.exe -t ${number of threads} -m ${minimum depth} ./blue_temp ${result file}

3.5.3) mono Blue.exe -m ${*k*-mer repetition depth} -t ${number of threads} -hp -good ${%*k*-mers} -variable -o ${result dir} ./blue_temp ${result file}

- ${k-mer size}: 21
- ${minimum depth}: the minimum depth needed before a k-mer will be loaded into the k-mers table (setting to 10)
- ${k-mer repetition depth}: a *k*-mer repetition depth used to distinguish between 'good' and 'bad' k-mers (setting to 50)

- ${%k-mers}: keeps reads that look to be ‘good’ after correction (setting to 80).

3.6 Pollux_indel and Fiona (pollux_fiona)

3.6.1) Pollux for indel error correction only

pollux -s false -n true -d true -h true -i ${input file} -k ${*k*-mer size} -o ${result dir}

- {-s false}: substitution correction is false
- {-n true}: insertion corrections is true
- {-d true}: deletion corrections is true
- ${*k*-mer size}: 31

3.6.2) Fiona for residual indel and substitution error correction

fiona -g ${genome size} -nt ${number of threads} ${input file} ${result file}

- ${genome size}: the genome size is the size of HBV reverse transcriptase region
- ${input file}: the fastq files is corrected only indels by Pollux
- ${result file}: specify a result fastq files of data

4. Optimization of Error correction parameter optimization. We tested whether the error-correction performance was associated with the value of *k*-mer (10, 21, and 31, respectively) for the methods of blue, pollux, and coral.

5. Error correction evaluation

The measure of gain, TP, FP, and FN were calculated using the 'compute-stats.py' script from the ECE Toolkit (http://aluru-sun.ece.iastate.edu/doku.php?id=ecr)[1]. In the calculation of TN, the count of the total bases from a fastq file was calculated using *readfq* (https://github.com/lh3/readfq).

python compute-stats.py -a ${pre-correction alignment-sam file} -m ${after-correction alignment-sam file} -o ${stats-output-file} -r ${number of reads} -g ${genome reference}

Supplementary tables

Table S1. The number of quality-trimmed reads and reads corrected by different algorithms

ID	Quality trimmed reads	pollux	blue	fiona	coral	karect	pollux_fiona
009	59,692	59,473	54,270	59,692	59,692	59,692	59,470
014	52,039	51,786	45,651	52,039	52,039	52,039	51,813
017	61,174	60,890	55,040	61,174	61,174	61,174	60,886
020	42,097	41,921	36,630	42,097	42,097	42,097	41,915
024	56,729	56,500	51,008	56,729	56,729	56,729	56,499
033	55,425	55,199	48,818	55,425	55,425	55,425	55,197
037	39,971	39,879	32,630	39,971	39,971	39,971	39,871
040	35,191	35,100	30,938	35,191	35,191	35,191	35,101
042	41,108	41,053	37,671	41,108	41,108	41,108	41,051
1005	40,081	39,951	38,704	40,081	40,081	40,081	39,950
1009	32,042	31,879	30,321	32,042	32,042	32,042	31,875
1014	36,652	36,556	35,771	36,652	36,652	36,652	36,558
1019	44,490	44,293	41,396	44,490	44,490	44,490	44,290
1024	57,965	57,555	55,124	57,965	57,965	57,965	57,562
1028	32,852	32,398	30,982	32,852	32,852	32,852	32,397
1034	42,626	42,500	40,545	42,626	42,626	42,626	42,500
1038	49,963	49,716	47,551	49,963	49,963	49,963	49,714
1041	40,300	39,843	37,185	40,300	40,300	40,300	39,840
1046	59,393	59,127	56,684	59,393	59,393	59,393	59,127

Table S2. The error rates (%) in the raw, quality-trimmed, and reads after error correction by different algorithms

ID	Raw reads	Quality trimmed reads	pollux	blue	fiona	coral	karect	pollux_fiona
009	0.6829	0.5160	0.0431	0.0503	0.2937	0.3352	0.4591	0.0198
014	0.7820	0.6150	0.1325	0.1089	0.4019	0.4136	0.5886	0.0986
017	0.6510	0.4960	0.0258	0.0607	0.2861	0.2963	0.4352	0.0065
020	0.8365	0.6229	0.0906	0.0527	0.3735	0.4127	0.5778	0.0611
024	0.7073	0.5369	0.0258	0.0548	0.3134	0.3278	0.4754	0.0102
033	0.7786	0.6031	0.0178	0.1370	0.3657	0.3584	0.5409	0.0036
037	0.7859	0.6099	0.0296	0.0776	0.3242	0.3427	0.5345	0.0137
040	0.7898	0.6135	0.0291	0.0558	0.3301	0.3634	0.5511	0.0101
042	0.7435	0.5558	0.0414	0.0632	0.2861	0.3088	0.4884	0.0234
1005	0.5131	0.4176	0.0396	0.0013	0.2032	0.2466	0.3555	0.0177
1009	0.5989	0.4937	0.0294	0.0011	0.2668	0.3374	0.4186	0.0114
1014	0.4374	0.3532	0.0209	0.0005	0.1577	0.2107	0.2933	0.0058
1019	0.4534	0.3681	0.0220	0.0012	0.1717	0.2344	0.2983	0.0059
1024	0.4443	0.3619	0.0200	0.0025	0.1897	0.2472	0.3151	0.0031
1028	0.4969	0.4146	0.0260	0.0011	0.2339	0.2898	0.3501	0.0104
1034	0.4152	0.3394	0.0399	0.0028	0.1581	0.2243	0.2852	0.0134
1038	0.4795	0.3931	0.1293	0.0237	0.2237	0.2596	0.3375	0.0857
1041	0.5087	0.4229	0.0696	0.0275	0.2345	0.2726	0.3657	0.0496
1046	0.4899	0.4099	0.0318	0.0029	0.2210	0.2540	0.3573	0.0164

Table S3. The proportion of the remained errors and mutated alleles after error-correction in simulated data with known variants.

	Pollux (%)	Blue (%)	Fiona (%)	Coral (%)	Karect (%)
Substitution errors	0.17±0.09	0.53±0.83	0.13±0.28	80.56±3.27	77.94±18.07
Rare variants	0.00±0.00	1.18±1.42	0.00±0.00	94.61±9.18	89.65±17.83
Low-frequency variants	0.03±0.05	2.85±0.33	100.06±0.11	72.30±4.87	99.35±0.34

Values are mean±sd. The number of mutated bases at a given position was extracted from TEF files. The error-corrected reads have a higher mapping rate, leading to >100% identification of mutated alleles (e.g., fiona).

Table S4. Error rates in the model 1 (a fixed substitution error rate and read number, and varied indels rates). We used a substitution error rate of 0.16985% from Laehnemann et al.[8]. The deletion error rate is 1.5 times of the insertion rate.

Insertion	Deletion	Substitution	Total
0.01	0.015	0.17	0.195
0.02	0.030	0.17	0.220
0.03	0.045	0.17	0.245
0.04	0.060	0.17	0.270
0.05	0.075	0.17	0.295
0.06	0.090	0.17	0.320
0.07	0.105	0.17	0.345
0.08	0.120	0.17	0.370

Table S5. Error rates in the model 2 (fixed indels error rates and read number, and varied substitution rates).

Insertion	Deletion	Substitution	Total
0.04	0.06	0.00	0.10
0.04	0.06	0.10	0.20
0.04	0.06	0.20	0.30
0.04	0.06	0.30	0.40
0.04	0.06	0.40	0.50
0.04	0.06	0.50	0.60
0.04	0.06	0.60	0.70
0.04	0.06	0.70	0.80

Table S6. Number of reads in the model 3 with a fixed substitution (0.17%) and fixed insertion and deletion (0.04% and 0.06%) error rate

Number of Read	Coverage
6,000	1,230
8,000	1,650
10,000	2,050
20,000	4,100
40,000	8,200
60,000	12,300

Supplementary Figures

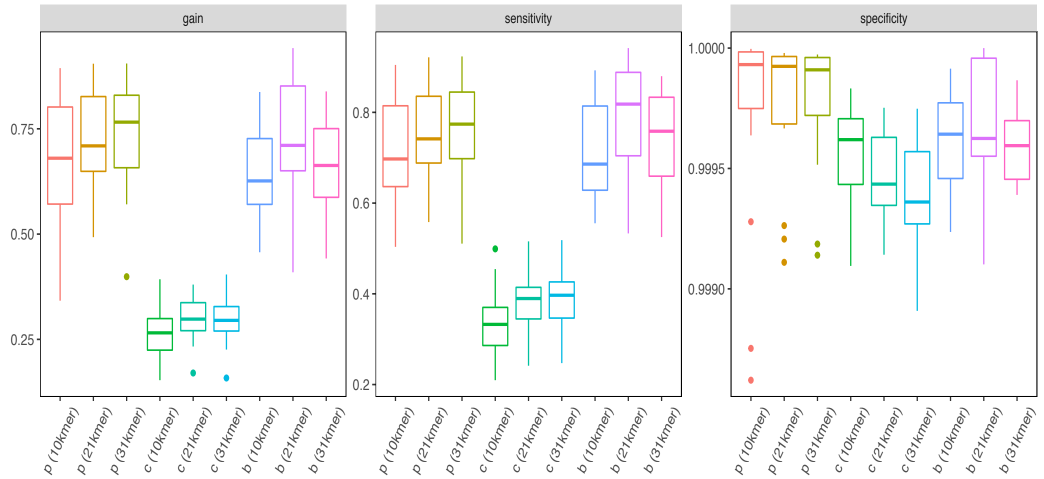

Fig. S1. The performance of different *k*-mer parameters for error-correction in the methods of pollux, coral and blue. (a). The measure of gain, (b). The sensitivity, and (c). The specificity. **(**p: pollux; b: blue; c: coral**)**

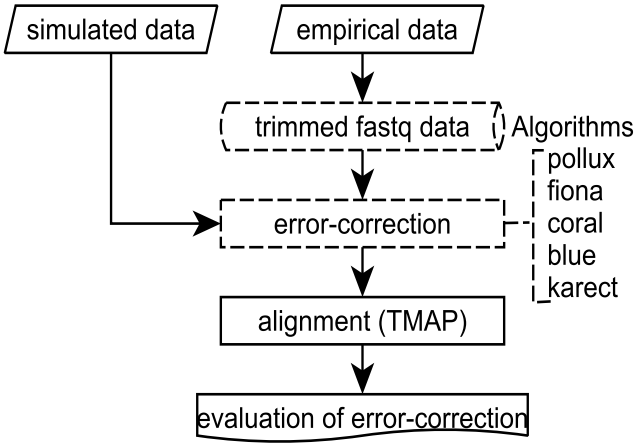

Fig. S2. A pipeline to process the empirical or simulated PGM data, including pre-processing, error correction, alignment, and assessment of error correction.

References

1. Yang, X., Chockalingam, S. P. & Aluru, S. A survey of error-correction methods for next-generation sequencing. *Briefings in bioinformatics* **14,** 56–66 (2013).
2. Caboche, S., Audebert, C., Lemoine, Y. & Hot, D. Comparison of mapping algorithms used in high-throughput sequencing: application to Ion Torrent data. *BMC Genomics* **15,** 1 (2014).
3. Allam, A., Kalnis, P. & Solovyev, V. Karect: accurate correction of substitution, insertion and deletion errors for next-generation sequencing data. *Bioinformatics* **31,** 3421–3428 (2015).
4. Schulz, M. H. *et al.* Fiona: a parallel and automatic strategy for read error correction. *Bioinformatics* **30,** i356–i363 (2014).
5. Marinier, E., Brown, D. G. & McConkey, B. J. Pollux: platform independent error correction of single and mixed genomes. *BMC Bioinformatics* **16,** 10 (2015).
6. Salmela, L. & Schröder, J. Correcting errors in short reads by multiple alignments. *Bioinformatics* **27,** 1455–1461 (2011).
7. Greenfield, P., Duesing, K., Papanicolaou, A. & Bauer, D. C. Blue: correcting sequencing errors using consensus and context. *Bioinformatics (2014)* **30,** 2723–2732 (2014).
8. Laehnemann, D., Borkhardt, A. & McHardy, A. C. Denoising DNA deep sequencing data-high-throughput sequencing errors and their correction. *Briefings in bioinformatics* bbv029 (2015).
